# Dehydroxymethylepoxyquinomicin, a novel nuclear factor-κB inhibitor, prevents the development of cyclosporine A nephrotoxicity in a rat model

**DOI:** 10.1186/s40360-020-00432-3

**Published:** 2020-08-12

**Authors:** Shinya Morita, Kazunobu Shinoda, Tadashi Yoshida, Masayuki Shimoda, Yoshihiko Kanno, Ryuichi Mizuno, Hidaka Kono, Hiroshi Asanuma, Ken Nakagawa, Kazuo Umezawa, Mototsugu Oya

**Affiliations:** 1 Department of Urology, Keio University School of Medicine, Tokyo, Japan; 2 Apheresis and Dialysis Center, Keio University School of Medicine, Tokyo, Japan; 3 Department of Nephrology, Toho University Faculty of Medicine, 7-5-23 Omorinishi Ota-ku, Tokyo, 143-0015 Japan; 4 Department of Pathology, Keio University School of Medicine, Tokyo, Japan; 5 Department of Nephrology, Tokyo Medical University, Tokyo, Japan; 6 Department of Urology, Tokyo Dental College Ichikawa General Hospital, Chiba, Japan; 7 Department of Molecular Target Medicine Screening, Aichi Medical University, Aichi, Japan

**Keywords:** Cyclosporine, Nephrotoxicity, NF-κB, NF-κB inhibitor

## Abstract

**Background:**

Cyclosporine A (CsA) is an essential immunosuppressant in organ transplantation. However, its chronic nephrotoxicity is an obstacle to long allograft survival that has not been overcome. Nuclear factor-κB (NF-κB) is activated in the renal tissue in CsA nephropathy. In this study, we aimed to investigate the effect of the specific NF-κB inhibitor, dehydroxymethylepoxyquinomicin (DHMEQ), in a rat model of CsA nephrotoxicity.

**Methods:**

We administered CsA (15 mg/kg) daily for 28 days to Sprague-Dawley rats that underwent 5/6 nephrectomy under a low-salt diet. We administered DHMEQ (8 mg/kg) simultaneously with CsA to the treatment group, daily for 28 days and evaluated its effect on CsA nephrotoxicity.

**Results:**

DHMEQ significantly inhibited NF-κB activation and nuclear translocation due to CsA treatment. Elevated serum urea nitrogen and creatinine levels due to repeated CsA administration were significantly decreased by DHMEQ treatment (serum urea nitrogen in CsA + DHMEQ vs CsA vs control, 69 ± 6.4 vs 113.5 ± 8.8 vs 43.1 ± 1.1 mg/dL, respectively, *p* < 0.0001; serum creatinine in CsA + DHMEQ vs CsA vs control, 0.75 ± 0.02 vs 0.91 ± 0.02 vs 0.49 ± 0.02 mg/dL, respectively, p < 0.0001), and creatinine clearance was restored in the treatment group (CsA + DHMEQ vs CsA vs control, 2.57 ± 0.09 vs 1.94 ± 0.12 vs 4.61 ± 0.18 ml/min/kg, respectively, p < 0.0001). However, DHMEQ treatment did not alter the inhibitory effect of CsA on urinary protein secretion. The development of renal fibrosis due to chronic CsA nephrotoxicity was significantly inhibited by DHMEQ treatment (CsA + DHMEQ vs CsA vs control, 13.4 ± 7.1 vs 35.6 ± 18.4 vs 9.4 ± 5.4%, respectively, p < 0.0001), and these results reflected the results of renal functional assessment. DHMEQ treatment also had an inhibitory effect on the increased expression of chemokines, monocyte chemoattractant protein-1, and chemokine (c-c motif) ligand 5 due to repeated CsA administration, which inhibited the infiltration of macrophages and neutrophils into the renal tissue.

**Conclusions:**

These findings suggest that DHMEQ treatment in combination therapy with CsA-based immunosuppression is beneficial to prevent the development of CsA-induced nephrotoxicity.

## Background

Although immunosuppression induced by calcineurin inhibitors (CNIs) has remarkably improved short-term graft survival in kidney transplantation, satisfactory long-term graft survival has yet to be obtained [1]. Although several lines of evidence have demonstrated that cyclosporine A (CsA), a CNI, elicits both acute and chronic nephrotoxicity, these problems remain unaddressed [2–5]. This unfavorable effect of CsA treatment has also been observed in patients treated with tacrolimus [6]. Multifactorial mechanisms underlie the histological damage due to CNI nephropathy [7], and nephrotoxicity induced by CsA in particular has been widely investigated.

Among the several molecular mechanisms directly affected by CsA, the activation of a key transcription factor, nuclear factor-κB (NF-κB), is critical. CsA is known to inhibit the NF-κB signaling that promotes the production of interleukin 2 in T cells [8, 9]. However, in tubular epithelial cells, CsA activates NF-κB and induces inflammation, eventually leading to tubulointerstitial fibrosis [10–12]. Transcriptomic analysis showed that CNIs upregulate the NF-κB signaling and its target genes, including monocyte chemoattractant protein-1 (MCP-1), Rantes, and interleukin 6 [13]. The authors found that CNI induced NF-κB activation through four different signaling pathways, the TLR4/Myd88/IRAK, JAK2/STAT3, TAK1/JNK/AP-1 pathways and the unfolded protein response, and investigated the effects of CNIs on each pathway [13]. Thus, NF-κB signaling regulation is the key to preventing the development of CNI nephropathy.

Our group has applied a newly designed inhibitor of NF-κB activation, dehydroxymethylepoxyquinomicin (DHMEQ), to several experimental models [14–17]. The mechanism of DHMEQ has been extensively studied. DHMEQ covalently binds to the specific cysteine residue of NF-κB components to inhibit their DNA binding [18, 19] and nuclear translocation [20, 21]. Drug activity of DHMEQ is highly NF-κB specific. DHMEQ has protective effects against renal ischemia reperfusion injury and unilateral ureteral obstruction injury [14, 16]. We have also shown that DHMEQ inhibits the activation of macrophages and the maturation of dendritic cells [15, 22]. Macrophage infiltration is one of the mechanisms by which chronic CsA nephrotoxicity develops [23]. Thus, DHMEQ is expected to act on both tubuloepithelial cells and immune cells. In the present study, we aimed to investigate whether CsA nephrotoxicity is ameliorated by DHMEQ treatment. We employed a rat CsA nephrotoxicity model, because DHMEQ is a preclinical drug and because rodent models with repeated CsA administration under low-sodium conditions have been shown to closely reproduce human CsA nephropathy [24].

## Methods

### Animals

8–10-week-old male Sprague-Dawley rats were purchased from CLEA Japan, Inc. (Tokyo, Japan). All rats were maintained under pathogen-free conditions in filter-topped cages with an automatic water system throughout the experiments. If rats underwent surgical treatment, each rat was housed in a single cage for 24 h. In other situations, 2–3 rats were housed in a single cage. All rats were cared for according to the Guidelines for Animal Experimentation of Keio University School of Medicine and current laws in Japan (Act on Welfare and Management of Animals). All animal experiments were approved by the Animal Ethics Committee at Keio University (approved number: 08061–7).

### Chronic CsA nephrotoxicity model

**Fig. 1:**
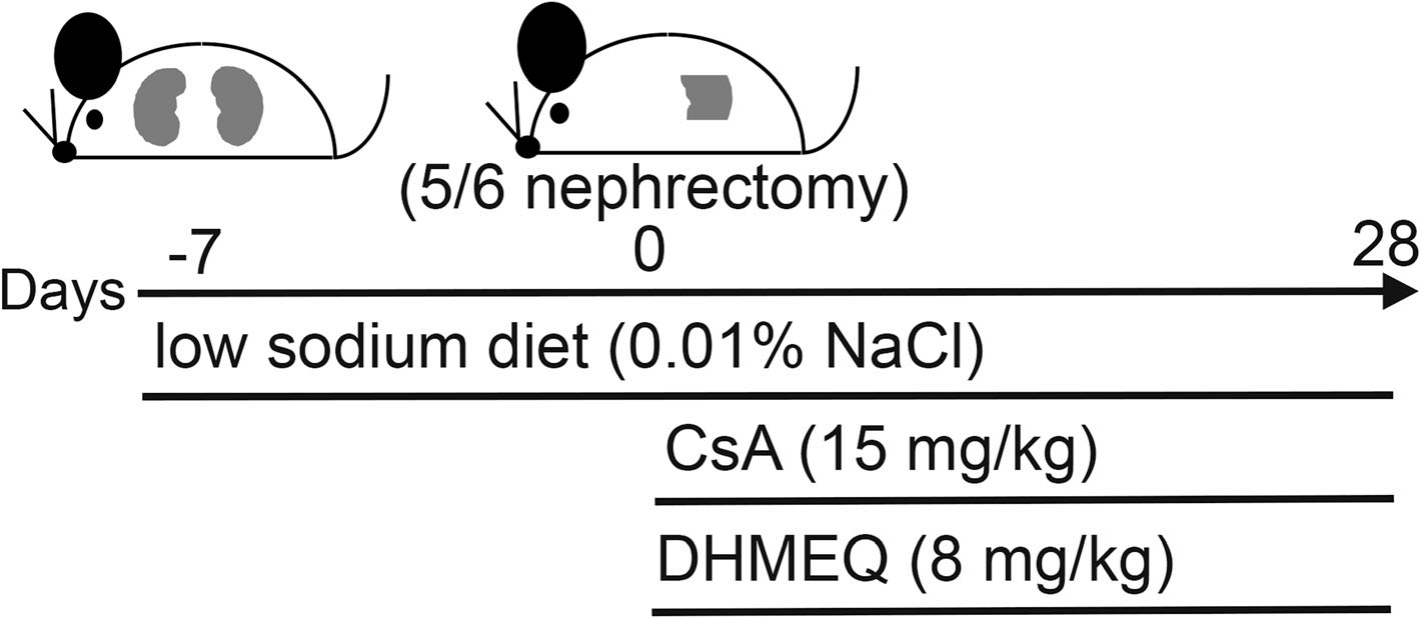
Schematic representation of the experimental design. Rats underwent 5/6 nephrectomy 7 days after the feeding with a low-sodium diet (0.01% NaCl) began. In the CsA treatment group (15 mg/kg), CsA administration began on the day of surgery and continued daily for 28 days. If the rats were cotreated with DHMEQ (8 mg/kg), DHMEQ administration began on the same day and continued daily for 28 days

Rats were fed a semisynthetic low-sodium diet (0.01% sodium) during the course of the experiment. Low-sodium conditions have been shown to augment the severity of CsA nephropathy in a rodent model by activating the renin-angiotensin system [24–26]. To decrease the number of nephrons, we performed 5/6 nephrectomy (right nephrectomy and segmental resection of the upper and lower poles of the left kidney) under inhalation anesthesia with 3% sevoflurane one week after beginning the feeding of the low-sodium diet. The rats were then treated with CsA (15 mg/kg) or 5% glucose by intraperitoneal administration daily for 28 days (Fig. 1). The dose of CsA was decided according to previous reports [24–26]. This dose is three-four times fold of that utilized in human clinical kidney transplantation [27].

### Drugs

CsA was obtained as a commercial product (Sandimmun, Novartis, Switzerland), dissolved in 5% glucose, and administered via intraperitoneal injection to each animal at a dose of 15 mg/kg.

DHMEQ was synthesized as previously described [28]. The purity was 95.3%, which was measured by HPLC by Tecno Chem CO., LTD. (Tokyo, Japan). DHMEQ was dissolved in DMSO to prepare a 10 mg/ml stock solution, diluted in olive oil, and administered via intraperitoneal injection to each animal at a dose of 8 mg/kg.

### Experimental protocol

The experimental protocol is shown in Fig. 1. All rats underwent 5/6 nephrectomy and were fed a low-sodium diet (0.01% sodium) as described above. Eighteen rats were randomly assigned and divided into three groups as follows: a control group treated with 5% glucose for 28 days (*n* = 6), CsA group treated with CsA (15 mg/kg daily) for 28 days (n = 6), and CsA + DHMEQ group treated with CsA (15 mg/kg daily) and DHMEQ (8 mg/kg daily) for 28 days (n = 6).

On day 28, we placed rats in metabolic cages for 24 h and collected urine and blood sample to measure urine volume, serum levels of urea nitrogen (UN) and creatinine (Cr), creatinine clearance (CCr), and urinary protein extraction. Finally, we administered inhalation anesthesia with 3% sevoflurane, removed kidney samples for further evaluation, and euthanized the animals by cutting abdominal aorta.

### Measurement of NF-κB (p65) DNA-binding activity

**Fig. 2:**
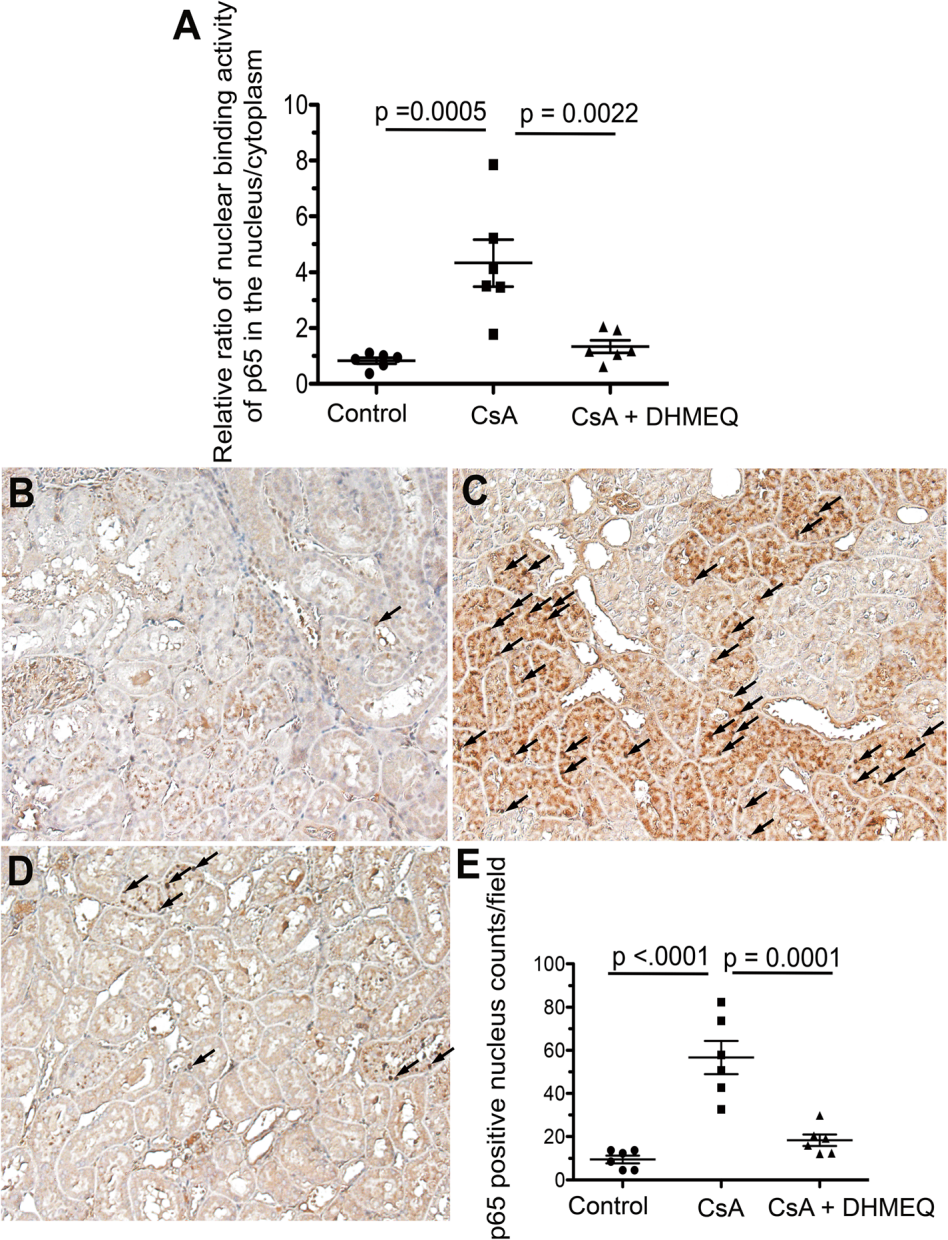
Analyses of the effect of DHMEQ treatment on NF-κB activity in CsA nephropathy. **a** DNA-binding activity of NF-κB (p65) in nuclear and cytoplasmic extracts, as determined by nonradioactive NF-κB-specific DNA-binding ELISA. The results are shown as the relative ratio of DNA-binding activity of NF-κB (p65) in the nucleus to that in the cytoplasm. **b** Representative immunohistochemical staining of p65 in the control. **c** Representative immunohistochemical staining of p65 in the CsA group. **d** Representative immunohistochemical staining of p65 in the CsA + DHMEQ group. All photos are magnified 100×. Arrows indicate nuclei positive for p65 staining. **e** The graph indicates the number of nuclei positively stained for p65 in each group. The circular, rectangular, and triangular dots represent the data in the control, CsA, and CsA + DHMEQ groups, respectively. The bars represent the mean values ± s.e.m.s.

Renal cortical tissue was homogenized, and nuclear and cytoplasmic extracts from the homogenized sample were prepared using nuclear and cytoplasmic extraction reagents (NE-PER, Thermo Fisher Scientific, Waltham, MA, USA). The DNA-binding activity of NF-κB (p65) was measured using a nonradioactive NF-κB-specific DNA-binding enzyme-linked immunosorbent assay (ELISA) kit (TransAM NF-κB p65 transcription factor assay kit, Active Motif, CA, USA), as previously described [16]. The results are shown as the relative ratio of NF-κB (p65) DNA-binding activity in the nucleus divided by that in the cytoplasm (binding activity in the nucleus / binding activity in the cytoplasm) (Fig. 2a).

### Histological assessment

The kidney samples were cut into halves and prepared for histological evaluation. One sample was fixed in 10% formalin and embedded in paraffin, and the other was embedded and frozen in OCT compound (Sakura Finetek USA Inc., Torrance, CA) before being stored at − 80 °C. The paraffin-embedded samples were sectioned into 4 μm sections and stained with Masson’s trichrome to evaluate the renal fibrosis area. The ratio of the renal fibrosis area in each region was calculated as follows. Ten areas of the cortex in each sample were randomly selected by a pathologist and captured digitally by light microscopy at 100× magnification. Image processing and analysis were performed by using ImageJ (NIH). The fibrosis area, which was defined as the collagen fiber-rich region, was stained blue, and the border of the fibrosis area was manually demarcated with ImageJ by an evaluator (Fig. 4g). The demarcated area was automatically quantitated, and the proportion of the fibrosis area in each field was calculated. If an essential structure of the kidney (e.g., glomeruli, tubules, peritubular capillaries, or vessels) was stained blue and seemed to be morphologically normal, this area was excluded from the fibrosis area. The pathologist and evaluator were blinded to information about the treatment of each sample.

### Immunohistochemistry

The paraffinized sections (4 μm thickness) were also processed for staining for NF-κB (p65) (clone F-6, mouse IgG1, Santa Cruz Biotechnology, CA, USA) and CD68 (clone ED1, mouse IgG1, Bio-Rad Laboratories, CA, USA). Cryosections (4 μm thickness) were also prepared using the frozen unfixed blocks described above. These cryosections were processed for granulocyte staining (clone HIS48, mouse IgM, Bio-Rad Antibodies, CA, USA).

The p65 staining protocol was as follows [29]. After deparaffinization in xylene, sections were rehydrated by incubation through a decreasing graded ethanol series (100%, changed 3 times, 5 min each; 95%, changed twice, 5 min each; and 70%, changed once, 5 min) and distilled water for 5 min. For antigen retrieval, the sections were soaked in unmasking solution (Vector Laboratories, CA, USA) and heated by microwave for 20 min. After endogenous peroxidase was blocked with 3% H_2_O_2_ for 10 min and nonspecific antibody (Ab) binding was blocked with 5% horse serum for 1 h, the sections were incubated with primary Ab (p65 F-6, 1:100 dilution) for 1 h at room temperature. After washing with phosphate-buffered saline, the sections were incubated with secondary Ab (biotinylated anti-mouse IgG, Vector Laboratories, CA, USA). Then, staining was detected using a Vectastatin ABC Kit (Vector Laboratories, CA, USA) and DAB solution. Nuclei were then counterstained with Mayer’s hematoxylin.

The CD68 staining protocol was as follows. The deparaffinization and rehydration steps were performed as described above. Antigen retrieval was performed using proteinase K for 15 min at room temperature. After endogenous peroxidase was blocked with 3% H_2_O_2_ for 10 min and nonspecific Ab binding was blocked with 6% skim milk for 15 min, the sections were incubated with primary Ab (ED1, 1:100 dilution) overnight at 4 °C. After washing with phosphate-buffered saline, the sections were incubated with peroxidase-conjugated secondary Ab (Histofine Simple Stain Rat Max- PO, Nichirei Co, Tokyo, Japan). Then, staining was detected using a DAB solution.

The granulocyte staining protocol was as follows. Each cryosection was dried and fixed in acetone for 10 min. After nonspecific Ab binding was blocked with Protein Block Serum-Free (DAKO, Agilent Pathology Solutions, CA, USA) for 10 min, the sections were incubated with primary Ab (HIS48, 1:20 dilution) for 1 h at room temperature. After washing with 0.05 mol/L Tris-HCl (pH 7.6) containing 0.15 mol/L NaCl, the endogenous peroxidase reaction was blocked with 0.3% H_2_O_2_/methanol for 30 min. After washing, the sections were incubated with biotinylated secondary antibody for 15 min at room temperature, and staining was detected using a Universal LSAB2 Kit/HRP (DAKO, Agilent Pathology Solutions, CA, USA) and DAB solution. Nuclei were then counterstained with Mayer’s hematoxylin.

Ten areas of the cortex in each sample were randomly selected by a pathologist and captured digitally by light microscopy at 100× magnification. One evaluator manually counted positively stained cells in each field. The pathologist and evaluator were blinded to information about the treatment of each sample.

### Real-time quantitative polymerase chain reaction (PCR)

The mRNA expression for MCP-1 and chemokine (c-c motif) ligand 5 (CCL5) was evaluated. We isolated total RNA from kidney samples by using RNAiso Plus kit (TaKaRa Bio, Shiga, Japan) and transcribed the RNA into cDNA. We performed real-time PCR by using a TaqMan Gene Expression Assay specific for each gene of interest and TaqMan Fast Universal PCR Master Mix on a StepOnePlus Real-Time PCR System (Applied Biosystems). Primer and probe sets were as follows: MCP-1 (Rn00580555_m1), CCL5 (Rn00579590_m1), and glyceraldehyde-3-phosphate dehydrogenase (GAPDH) (Rn01775763_g1) as an endogenous control. Relative quantification was performed by comparing the threshold cycle values of samples with those of serially diluted standards. Each result was normalized to GAPDH. The results are ratios (mean values ± s.e.m.s) of levels in the CsA nephropathy and DHMEQ groups to those in the control group, with average values in the control group set as 1.0.

### Statistical analysis

Data were collected and analyzed from all animals (100%) in each group. Results are given as the mean ± s.e.m. Variables among groups were compared using analysis of variance (ANOVA), with *p* < 0.05 indicating a significant difference. When the ANOVA test indicated significance, Tukey-Kramer’s test was used as a post hoc test. Only significant *p* values are shown in each figure. These analyses were performed with dedicated statistical software (JMP v13.2.0, SAS Institute, Inc., Cary, NC, USA), and statistical figures were prepared using GraphPad Prism v5.0 (GraphPad Software, San Diego, CA, USA).

## Results

### DHMEQ treatment significantly inhibited the nuclear translocation of p65 in rat kidney tissue

The major form of NF-κB is a heterodimer (p65/p50) that is inactivated when bound to IκB in the cytoplasm; this heterodimer is translocated to the nucleus after the phosphorylation and degradation of IκB via activation signals from the cell surface membrane [30]. DHMEQ has been shown to inhibit nuclear translocation of the activated NF-κB heterodimer (p65/p50) [17, 20]. Therefore, we investigated whether DHMEQ treatment inhibited the nuclear translocation of p65 in a CsA nephropathy model. We did not observe any adverse events (e.g. phenotypical or behavioral abnormalities) on animals in each group due to drug administration.

We separated the nuclear and cytoplasmic proteins from digested kidney samples and evaluated the activity of NF-κB in the nuclear and cytoplasmic fractions by ELISA. As suggested in several previous reports [11, 12], NF-κB activation and the nuclear translocation of p65 in the kidneys of rats treated with CsA were significantly increased compared with those in the control rats (control vs CsA, 0.83 ± 0.11-fold vs 4.33 ± 0.84-fold increase, relative ratio of p65 DNA-binding activity in the nucleus to that in the cytoplasm, respectively, *p* = 0.0005, Fig. 2a). However, the nuclear translocation of p65 in the rat kidney was significantly inhibited by cotreatment with DHMEQ compared with CsA monotherapy (CsA + DHMEQ vs CsA, 1.34 ± 0.23-fold vs 4.33 ± 0.84-fold increase, relative ratio of p65 DNA-binding activity in the nucleus to that in the cytoplasm, respectively, *p* = 0.0022, Fig. 2a). There was no significant difference of p65 DNA-binding activity between the control and the CsA + DHMEQ group (control vs CsA + DHMEQ, 0.83 ± 0.11-fold vs 1.34 ± 0.23-fold, respectively, *p* = 0.7623, Fig. 2a).

We also evaluated the effect of NF-κB activation on the histology by immunohistochemical staining. In accordance with the results obtained by ELISA, the nuclear translocation of p65 was increased in rats treated with CsA compared with control untreated rats (control vs CsA, 9.5 ± 1.8 vs 56.7 ± 7.7 nuclear counts/field, respectively, *p* < 0.0001, Fig. 2b, c, e). The affected area was mostly in the tubular epithelial cells (Fig. 2c). However, DHMEQ treatment effectively inhibited the nuclear translocation of p65 due to the administration of CsA (CsA + DHMEQ vs CsA, 18.3 ± 2.7 vs 56.7 ± 7.7 nuclear counts/field, respectively, *p* = 0.0001, Fig. 2c, d, e). There was no significant difference of the nuclear translocation of p65 between the control and the CsA + DHMEQ group (control vs CsA + DHMEQ, 9.5 ± 1.8 vs 18.3 ± 2.7, respectively, *p* = 0.4198, Fig. 2b, d, e).

### DHMEQ treatment ameliorated renal function deterioration by CsA

**Fig. 3:**
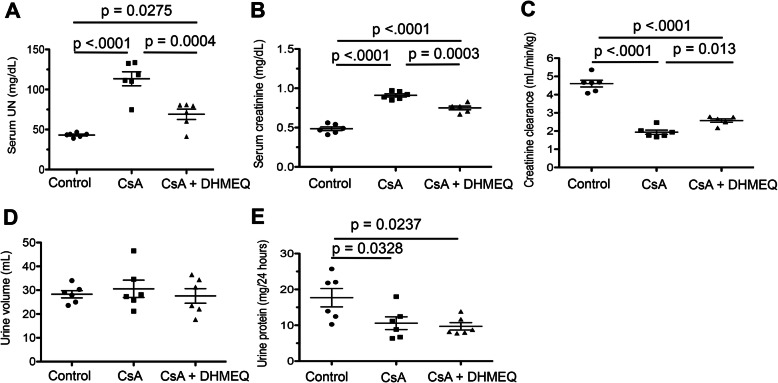
Analyses of renal function. Comparison of the serum UN level (**a**), serum creatinine level (**b**), creatinine clearance (**c**), urine volume (**d**), and urinary protein extraction (**e**) in each group. The circular, rectangular, and triangular dots represent the data in the control, CsA, and CsA + DHMEQ groups, respectively. The bars represent the mean values ± s.e.m.s.

The growth of the rats in each group that was assumed from body weight increases from the baseline and to the day of euthanasia was not statistically different in each group (Δ weight in control vs CsA vs CsA + DHMEQ, 61.7 ± 38.9 vs 26.2 ± 41.9 vs 15.8 ± 21.8 g, *p* = 0.0931 by ANOVA, supplementary Table 1). Repeated administration of CsA (15 mg/kg/day for 28 days) and low-sodium conditions caused the deterioration of renal function in a 5/6 nephrectomized rat model. Serum UN levels in the CsA nephropathy group were significantly increased compared with those in the control group (control vs CsA, 43.1 ± 1.1 vs 113.5 ± 8.8 mg/dL, respectively, *p* < 0.0001, Fig. 3a). The serum Cr level was also increased in the CsA nephropathy group compared with the control group (control vs CsA, 0.49 ± 0.02 vs 0.91 ± 0.02 mg/dL, respectively, *p* < 0.0001, Fig. 3b). We calculated the CCr and normalized the results by body weight (kg). Normalized CCr in the CsA nephropathy group was decreased compared with the control group (control vs CsA, 4.61 ± 0.18 vs 1.94 ± 0.12 ml/min/kg, respectively, p < 0.0001, Fig. 3c).

However, DHMEQ treatment significantly ameliorated renal function deterioration caused by repeated CsA administration. Serum UN levels in the CsA + DHMEQ group were significantly decreased compared with those in the CsA group (CsA + DHMEQ vs CsA, 69 ± 6.4 vs 113.5 ± 8.8 mg/dL, respectively, *p* = 0.0004, Fig. 3a). The serum Cr level in the CsA + DHMEQ group was also significantly decreased compared with that in the CsA group (CsA + DHMEQ vs CsA, 0.75 ± 0.02 vs 0.91 ± 0.02 mg/dL, respectively, *p* = 0.0003, Fig. 3b). In addition, CCr was significantly increased in the CsA + DHMEQ group compared with the CsA group (CsA + DHMEQ vs CsA, 2.57 ± 0.09 vs 1.94 ± 0.12 ml/min/kg, respectively, *p* = 0.013, Fig. 3c). However, DHMEQ treatment did not completely restore renal function to the control level (serum UN, Cr, and CCr in control vs CsA + DHMEQ; 43.1 ± 1.1 vs 69 ± 6.4 mg/dL, *p* = 0.0275; 0.49 ± 0.02 vs 0.75 ± 0.02 mg/dL, *p* < 0.0001; 4.61 ± 0.18 vs 2.57 ± 0.09 ml/min/kg, p < 0.0001; respectively, Fig. 3A, B, and C).

In contrast, the urine volume in each group was not significantly different (control vs CsA vs CsA + DHMEQ, 28.3 ± 1.5 vs 30.6 ± 3.6 vs 27.6 ± 3.1 ml, Fig. 3d). Interestingly, urinary protein extraction was significantly decreased in the CsA nephropathy group compared with the control group (control vs CsA, 17.7 ± 2.6 vs 10.6 ± 1.8 mg/24 h, respectively, *p* = 0.0328, Fig. 3e). DHMEQ treatment did not offset the inhibitory effect of urinary protein extraction due to CsA (CsA + DHMEQ vs CsA, 9.7 ± 1.0 vs 10.6 ± 1.8 mg/24 h, *p* = 0.9255; control vs CsA + DHMEQ, 17.7 ± 2.6 vs 9.7 ± 1.0 mg/24 h, *p* = 0.0237; respectively, Fig. 3e).

### DHMEQ treatment significantly inhibited the development of renal fibrosis due to CsA

**Fig. 4:**
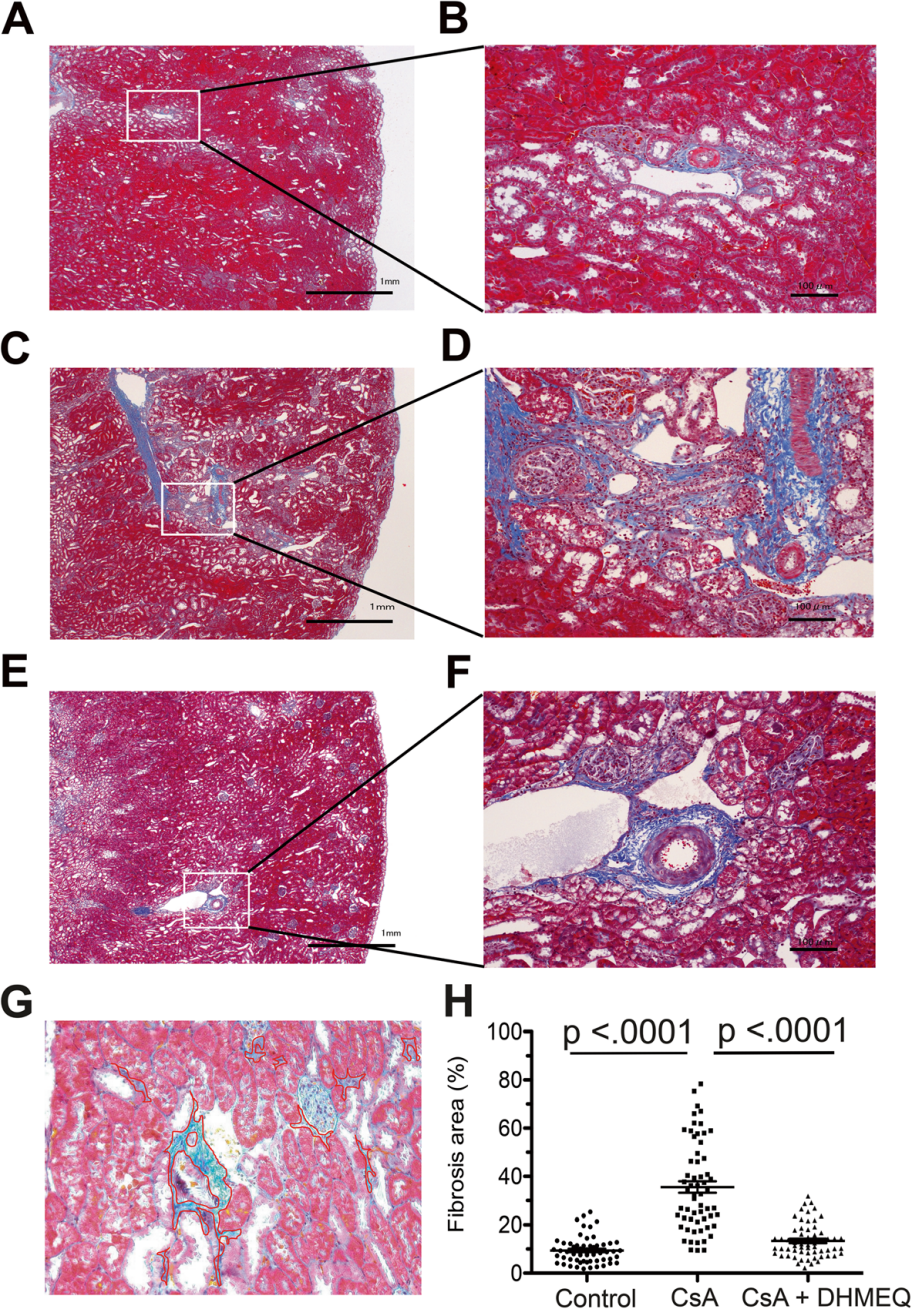
Evaluation of the renal fibrosis area. **a**, **c**, and **e** show representative Masson’s trichrome staining in the control, CsA, and CsA + DHMEQ groups, respectively. **a**, **c**, and **e** are magnified 20×. The areas highlighted in the small boxes in the left panels (**a**, **c**, **e**) are shown in the right panels (**b**, **d**, **f**) at a magnification of 100×. **g** We demarcated the border (red line in the photo) of the blue stained area in the interstitium and excluded essential kidney structures (e.g., the glomeruli, tubules, peritubular capillaries, or vessels). The border was drawn manually with ImageJ software. **h** The graph indicates the percentage of the fibrosis area in each group. The circular, rectangular, and triangular dots represent the data in the control, CsA, and CsA + DHMEQ groups, respectively. The bars represent the mean values ± s.e.m.s.

Next, we investigated whether the deterioration of renal function was associated with renal tissue fibrosis among the three groups. Surgical treatment with 5/6 nephrectomy (control), which was intended to reduce the number of nephrons, did not affect renal fibrosis formation (Fig. 4a, b). In contrast, renal fibrosis developed in the kidneys of rats treated with CsA (Fig. 4c, d). Typical striped renal fibrosis from the corticomedullary boundary to the surface of the cortex was observed (Fig. 4c). The renal fibrosis area was significantly increased in the CsA group compared with the control group (control vs CsA, 9.4 ± 5.4 vs 35.6 ± 18.4%, respectively, *p* < 0.0001, Fig. 4h). However, renal fibrosis formation was remarkably inhibited by DHMEQ treatment (Fig. 4e, f). The renal fibrosis area was significantly decreased in the CsA + DHMEQ group compared with the CsA group (CsA + DHMEQ vs CsA, 13.4 ± 7.1 vs 35.6 ± 18.4%, respectively, p < 0.0001, Fig. 4h). There was no significant difference in the renal fibrosis area between the control and CsA + DHMEQ (control vs CsA + DHMEQ, 9.4 ± 5.4 vs13.4 ± 7.1%, respectively, *p* = 0.157, Fig. 4h).

### DHMEQ treatment significantly inhibited inflammatory cell infiltration

**Fig. 5:**
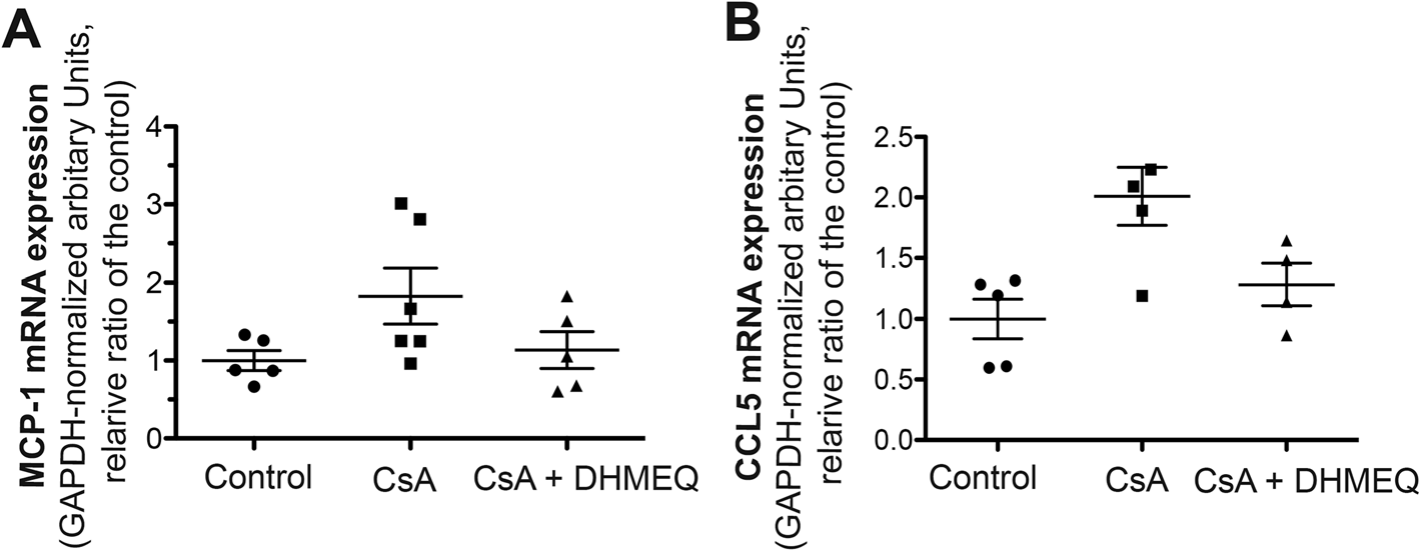
Real-time PCR assessment of chemokines in renal tissue. The graphs indicate the mRNA expression of MCP-1 (**a**) and CCL-5 (**b**). Each result was normalized to GAPDH as an endogeneous control. The results are ratios (mean values ± s.e.m.s) of levels in the CsA nephropathy and DHMEQ groups to those in the control group, with average values in the control group set as 1.0. The circular, rectangular, and triangular dots represent the data in the control, CsA, and CsA + DHMEQ groups, respectively. The bars represent the mean values ± s.e.m.s.

We further evaluated inflammatory cell infiltration in the kidneys of rats in the three groups. First, we evaluated the transcription of chemokines, MCP-1 and CCL5 in each group. MCP-1 mRNA expression levels in the CsA group were higher than those in the control group (control vs CsA, 1.00 ± 0.13 vs 1.82 ± 0.35, Fig. 5a). However, MCP-1 mRNA expression levels in the CsA + DHMEQ group were lower than those in the CsA group, although this difference was not statistically significant (CsA + DHMEQ vs CsA, 1.14 ± 0.24 vs 1.82 ± 0.35, Fig. 5a). The same tendency was observed for CCL5 (control vs CsA vs CsA + DHMEQ, 1.00 ± 0.10 vs 1.98 ± 0.42 vs 1.28 ± 0.17, Fig. 5b).

**Fig. 6:**
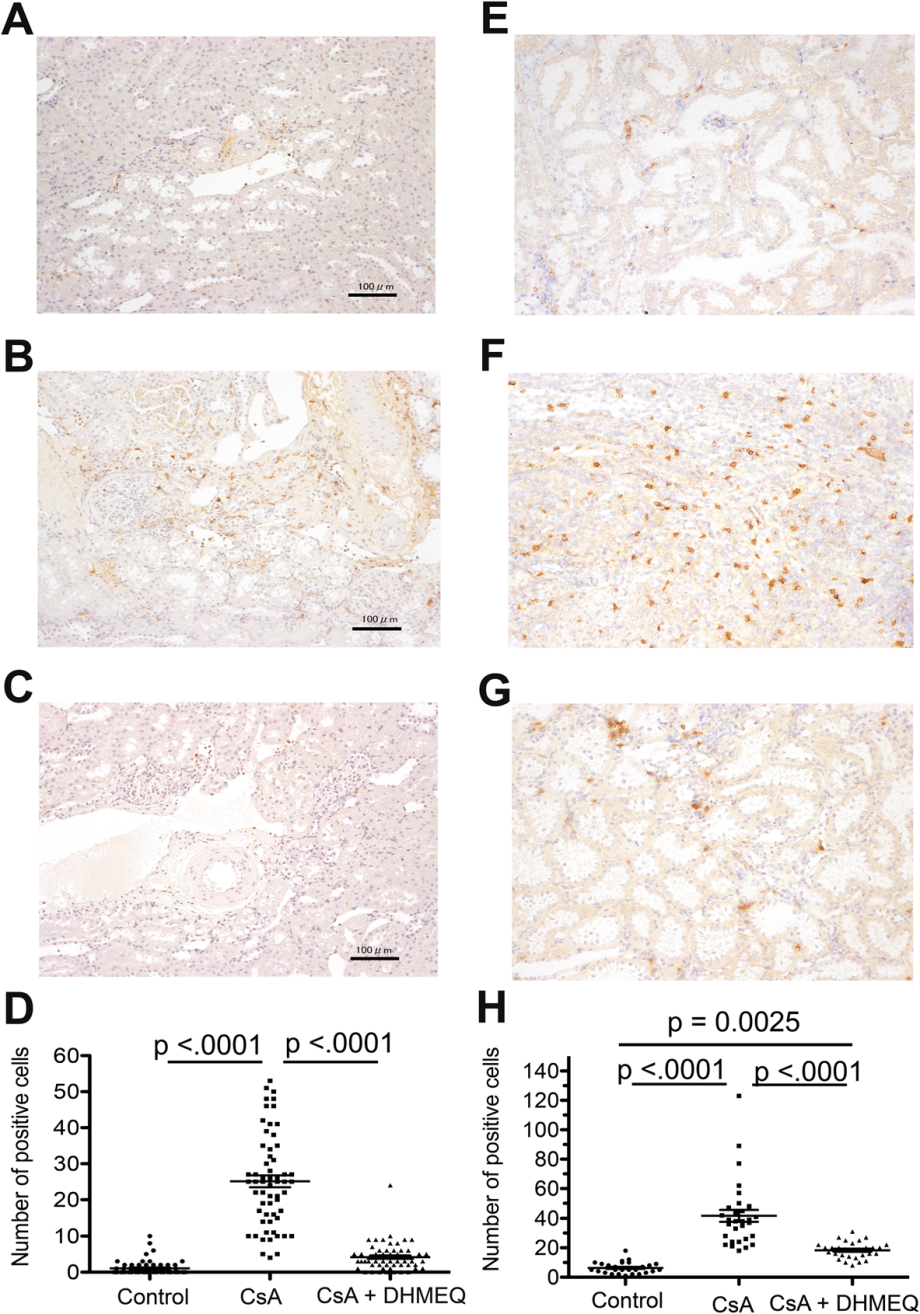
Infiltration of macrophages and granulocytes to renal tissue. **a**, **b**, and **c** show representative ED1 staining (macrophages) in the control, CsA, and CsA + DHMEQ groups, respectively. **e**, **f**, and **g** show representative HIS48 staining (granulocytes) in the control, CsA, and CsA + DHMEQ groups, respectively. **d** The graph indicates the number of cells positive for ED1 staining in each group. **h** The graph indicates the number of cells positive for HIS48 staining in each group. The circular, rectangular, and triangular dots represent the data in the control, CsA, and CsA + DHMEQ groups, respectively. The bars represent the mean values ± s.e.m.s.

Next, we investigated whether these changes in chemokine expression were associated with inflammatory cell infiltration in the renal tissue. Macrophage (ED1-positive cells) infiltration in the CsA group was significantly increased compared with that in the control group (control vs CsA, 1.1 ± 0.26 vs 25.1 ± 1.65 positive cells/field, respectively, *p* < 0.0001, Fig. 6a, b, d). However, macrophage infiltration in the CsA + DHMEQ group was significantly decreased compared with that in the CsA group (CsA + DHMEQ vs CsA, 4.2 ± 0.48 vs 25.1 ± 1.65 positive cells/field, respectively, p < 0.0001, Fig. 6b, c, d), and there was no significant difference of macrophage infiltration between the control and CsA + DHMEQ (control vs CsA + DHMEQ, 1.1 ± 0.26 vs 4.2 ± 0.48 positive cells/field, respectively, *p* = 0.0751, Fig. 6a, c, d). These findings were in accordance with the changes in MCP-1 expression.

We subsequently evaluated granulocyte infiltration in the renal tissue. Granulocyte (HIS48 positive cells) infiltration in the CsA group was significantly increased compared with that in the control group (control vs CsA, 6.3 ± 0.68 vs 41.8 ± 4.1 positive cells/field, respectively, p < 0.0001, Fig. 6e, f, h). In contrast, granulocyte infiltration was significantly decreased in the CsA + DHMEQ group compared with the CsA group (CsA + DHMEQ vs CsA, 18.4 ± 1.01 vs 41.8 ± 4.1 positive cells/field, respectively, p < 0.0001, Fig. 6f, g, h), although DHMEQ treatment did not completely inhibit granulocyte infiltration to the control level (control vs CsA + DHMEQ, 6.3 ± 0.68 vs 18.4 ± 1.01 positive cells/field, respectively, *p* = 0.0025, Fig. 6e, g, h).

## Discussion

In this study, we showed that DHMEQ treatment significantly ameliorated the deterioration of renal function and renal fibrosis due to CsA nephrotoxicity. The inhibition of macrophage and granulocyte infiltration by DHMEQ probably contributed to the protection of the kidney against histopathological and functional damages due to the administration of CsA.

The NF-κB transcriptional signaling was activated in the renal tissue over the course of CsA-induced renal damage, as several previous studies have suggested [11–13]. The immunohistochemical results in the present study revealed that the activated p65 translocated to the nuclei in mainly tubular epithelial cells. Renal histological injury is likely to be caused by the indirect effects of CsA; typical finding is prolonged arteriolar vasoconstriction, leading to local hypoxia, ischemia, and the production of free radicals or reactive oxygen species (ROS) [31–33]. More recently, direct cellular damage due to CsA has been demonstrated. In vitro studies revealed that CsA directly affects tubular epithelial cells, leading to the secretion of ROS, transforming growth factor-β, and procollagen and the activation of apoptotic genes [34–39]. Several studies have suggested that the NF-κB is the key to the mechanisms of the direct toxic effect of CsA in renal damage [11, 13]. The importance of NF-κB inhibition in an adriamycin-induced nephropathy model has also been suggested [40]. The findings in this study are in accordance with those reported in previous studies [11, 13, 39].

The findings of this study suggest that treatment with DHMEQ, a highly specific inhibitor of NF-κB activation, can interfere with CsA-induced nephrotoxicity. CsA, the use of which changed conventional immunosuppressive protocol based on azathioprine and prednisolone in the late 1970s [41], dramatically improved the outcome of organ transplantation and remains a key drug in current immunosuppressive protocols. However, in some cases, chronic CsA immunosuppression leads to irreversible renal damage due to the non-immunological molecular mechanisms described above. Chronic CsA nephropathy ultimately results in renal fibrosis that manifests as striped fibrosis in the medullary ray [24]. It is difficult to ameliorate this severe chronic change, and no effective treatments are currently available. This study showed that DHMEQ effectively improved renal histological damage due to the repeated administration of CsA. DHMEQ is a potential drug against CsA-induced nephrotoxicity. Because DHMEQ is a preclinical drug, we had no choice to use a rat CsA nephrotoxicity model to investigate the efficacy of DHMEQ, and we considered that the sample size for each group was essential for the statistical analyses. Moreover, DHMEQ has been shown to have immunosuppressive effects on immune cells, including macrophages and dendritic cells [15, 22]. Because of its wide range effects, DHMEQ has potential application in organ transplantation. Furthermore, CsA is known to inhibit urinary protein extraction [42], which was not altered by DHMEQ treatment (Fig. 3e).

Several studies have suggested that macrophage infiltration contributes to the development of CsA-induced renal fibrosis [23, 43]. Young et al. showed that macrophage influx preceded the development of renal interstitial fibrosis and afferent arteriolar hyalinosis in a rat CsA nephropathy model [43]. They demonstrated that the fibrosis score remained low until day 10, and the highest fibrosis score was detected on day 35 after the experiments began. In contrast, substantial macrophage infiltration was detected from 5 to 10 days after the experiments began and increased thereafter until day 35. Carlos et al. showed that macrophage depletion attenuated tubulointerstitial fibrosis and renal functional damage associated with CsA nephropathy in a rat model [23]. Two mechanisms may be related to the DHMEQ-induced inhibition of macrophage infiltration reported in this study. One mechanism, an indirect effect of DHMEQ, is a reduction in MCP-1 secretion (Fig. 5a). MCP-1, also known as chemokine (c-c motif) ligand 2 (CCL2), is one of the major mediators of chemotaxis and macrophage activation [44]. Many previous reports have suggested that CCL2 expression in the renal tissue of animal models is closely related to the development of renal fibrosis due to CNI toxicity [10, 45, 46]. The other mechanism is a direct effect of DHMEQ. Suzuki and Umezawa showed that DHMEQ inhibited macrophage activation and phagocytosis [22]. In addition, they showed that DHMEQ significantly inhibited the production of inducible NO synthase, NO, IL-6, TNF-α, and prostaglandin E2 in LPS-activated murine macrophages. DHMEQ also inhibited the differentiation of stimulated macrophages. However, there has been little research on the relationship between granulocytes and the development of CsA-induced renal fibrosis. We demonstrated that the infiltration of both granulocytes and macrophages may be related to the formation of CsA-induced renal fibrosis. In short, this study showed that both direct and indirect effects of DHMEQ presumably contributed to its interference of the activation of innate immunity due to CsA nephrotoxicity.

NF-κB inhibition is not the only solution to combat CsA nephrotoxicity, and DHMEQ treatment did not completely offset the histopathological damage and deterioration in renal function caused by CsA treatment. Other important factors in CsA nephrotoxicity are upregulation of the vasoconstrictor endothelin or the renin-angiotensin system and downregulation of the vasodilators prostaglandin E2 or NO [47–50]. A recent report suggested that the neutralization of high-mobility group box 1, a nuclear transcriptional factor, ameliorated chronic CsA nephrotoxicity [51]. The JAK2/STAT signaling pathway is also a key to the development of CsA nephropathy [52]. However, a benefit of DHMEQ treatment in CsA nephropathy is its additional immunosuppressive effects. DHMEQ has good potential for application in organ transplantation.

## Conclusion

This study showed that DHMEQ treatment specifically inhibited NF-κB activation and alleviated functional and histological damages in the kidneys of rats exposed to CsA treatment. Treatment with DHMEQ may be a solution to CsA nephrotoxicity in organ transplant therapy.

## Supplementary information

**Additional file 1.**

## Data Availability

The data that support the findings of this study are available from the corresponding author upon reasonable request.

## Abbreviations

CNIs: Calcineurin inhibitors
CsA: Cyclosporine A
NF-κB: Nuclear factor-κB
MCP-1: monocyte chemoattractant protein-1
DHMEQ: Dehydroxymethylepoxyquinomicin
UN: Urea nitrogen
Cr: Creatinine
CCr: Creatinine clearance
PCR: Polymerase chain reaction
CCL5: Chemokine (c-c motif) ligand 5
ELISA: Enzyme-linked immunosorbent assay
Ab: Antibody
GAPDH: Glyceraldehyde-3-phosphate dehydrogenase
ANOVA: Analysis of variance
ROS: Reactive oxygen species
CCL2: Chemokine (c-c motif) ligand 2
