# Supplementary material for: Dehydroxymethylepoxyquinomicin, a novel nuclear factor-κB inhibitor, prevents the development of cyclosporine A nephrotoxicity in a rat model
Source: BMC Pharmacol Toxicol. 2020 Aug 12;21:60. doi: 10.1186/s40360-020-00432-3 (PMC7424678; doi:10.1186/s40360-020-00432-3)
Supplement: Supplementary file 1 — Additional file 1. [file 40360_2020_432_MOESM1_ESM.docx]

Supplementary table 1: Body weight changes of the employed rats

| Groups | Animal # | Body weight (g) | | Δ body weight (g) (Day28-Day0) |
| --- | --- | --- | --- | --- |
|  |  | Day0 | Day28 |  |
| Control | 1 | 231.3 | 316.9 | 85.2 |
|  | 2 | 248.8 | 308.3 | 59.4 |
|  | 3 | 254.4 | 330.2 | 75.8 |
|  | 4 | 325.3 | 311 | -14.3 |
|  | 5 | 272.3 | 363.8 | 91.5 |
|  | 6 | 256.3 | 329.4 | 73.1 |
| CsA | 7 | 331.9 | 299.3 | -32.7 |
|  | 8 | 243.6 | 307.4 | 63.8 |
|  | 9 | 249.2 | 313.8 | 64.6 |
|  | 10 | 237.7 | 276.3 | 38.6 |
|  | 11 | 245 | 286.7 | 41.7 |
|  | 12 | 249.7 | 230.6 | -19.1 |
| CsA + DHMEQ | 13 | 308.8 | 3331.3 | 22.5 |
|  | 14 | 291.2 | 320.6 | 29.4 |
|  | 15 | 256 | 244.2 | -11.8 |
|  | 16 | 246.8 | 253.3 | 6.5 |
|  | 17 | 233.3 | 281.6 | 48.3 |
|  | 18 | 296.9 | 297 | 0.1 |
